# Editorial: Targeting neuroinflammation for novel therapeutics in neurodegenerative diseases

**DOI:** 10.3389/fphar.2025.1602495

**Published:** 2025-04-25

**Authors:** Pukar Khanal, Rupesh Chikhale, Jatin Machhi

**Affiliations:** ^1^ Emory University, Atlanta, GA, United States; ^2^ Department of Pharmaceutical and Biological Chemistry, School of Pharmacy, University College London, London, United Kingdom; ^3^ University of Nebraska Medical Center, Omaha, NE, United States

**Keywords:** Amyotrophic Lateral Sclerosis, Alzheimer’s disease, computational pharmacology, nanoengineering, Parkinson’s disease, ethnopharmacology

## Introduction

Neuroinflammation has emerged as a pivotal driver in the initiation and advancement of neurodegenerative disorders, including Alzheimer’s (AD) and Parkinson’s disease (PD). Historically, anti-inflammatory therapies faced critical hurdles in clinical translation which were limited by challenges such as blood-brain barrier (BBB) penetration, off-target effects, and the complex, multi-faceted nature of neuroimmune signaling. However, recent breakthroughs are reshaping this landscape. Advances in decoding inflammatory cascades and innovations in drug delivery systems enable precise modulation of brain immunity. Concurrently, the field is shifting from single-target drugs to integrative strategies, e.g., repurposed immunomodulators, synergistic natural compounds, and computational models that predict multi-target drug interactions. This Research Topic reflects a transformative focus—moving beyond palliative care to address the root causes of neurodegeneration. By harmonizing cutting-edge technology, cross-disciplinary research, and patient-specific biomarker profiling, these approaches aim to achieve durable disease modification, heralding a new era in neurodegenerative disease therapeutics.

## Bridging acute and chronic neurological disorders through non-invasive and metabolic interventions

The non-invasive neuromodulation has gained traction as an essential pillar for neuroprotection, offering distinct yet complementary mechanisms to combat neuroinflammation. In a pioneering study by Meng et al., the therapeutic potential of short-chain fatty acids (SCFAs) is contrasted with intermittent theta burst stimulation (iTBS) in treating delayed encephalopathy following acute carbon monoxide poisoning. Their work reveals a temporal dichotomy—iTBS elicits immediate cognitive improvements and synaptic plasticity, while SCFAs exert sustained neuroprotection by dampening pro-inflammatory cytokines and restoring glutamatergic/GABAergic balance. This dichotomy provokes a critical hypothesis—Could sequential or combined application of iTBS and SCFAs harmonize acute functional recovery with long-term anti-inflammatory resilience, optimizing outcomes in time-sensitive neuropathologies? Shifting focus to chronic neurodegeneration, Yang et al. explore the broad effects of a traditional herbal formulation, Xixin Decoction (XXD) in AD. Their findings extend beyond conventional amyloid-beta (Aβ) targeting, demonstrating XXD efficacy to repair BBB integrity by upregulating tight junction proteins (claudin-5, occludin)—and rebalancing the RAGE/LRP1 axis. By concurrently mitigating neurovascular dysfunction, suppressing NLRP3 inflammasome activation, and enhancing glymphatic drainage, XXD addresses AD multifactorial etiology. This polypharmacological approach underscores the XXD value in tackling complex neuroinflammatory cascades, positioning it as a viable adjunct to monoclonal antibody therapies, which often lack BBB penetrance or holistic immunomodulation.

Together, these studies highlight the evolving landscape of neuroinflammation therapeutics, where metabolic interventions and neuromodulation offer stratified benefits. Future research may prioritize combinatorial trials—e.g., pairing iTBS with SCFA supplementation in acute brain injury or integrating XXD with anti-Aβ immunotherapies to test for synergistic efficacy in AD. As the field advances, personalized sequencing of these modalities may emerge as a paradigm for bridging acute recovery and chronic disease modification.

## Harnessing nature’s pharmacy: innovations in bioactive compounds for neuroprotection

The resurgence of natural compounds as multi-targeted neurotherapeutics reflects a paradigm shift toward harmonizing traditional medicine with cutting-edge delivery technologies. Revankar et al. exemplifies this synergy by engineering an intranasal quercetin-loaded niosomal gel to surmount the flavonoid’s notorious bioavailability challenges in PD. The formulation not only enhances brain delivery but also demonstrates striking motor recovery and attenuation of oxidative stress, underscoring how advanced drug delivery systems can resurrect poorly bioavailable phytochemicals. In cerebral ischemia-reperfusion injury, Zhou et al. redefine neuroprotection with Jie-Du-Huo-Xue decoction (JDHXD). By independently suppressing both pyroptosis and autophagy, JDHXD uniquely disrupts the pathological interplay between inflammatory cell death and dysregulated protein clearance. The discovery of compartment-specific GSDMD localization in infarcted tissue not only challenges dogma but also opens avenues for spatially targeted stroke therapies. Next, epilepsy research illustrates the transformative potential of bioenhancement strategies. A review by Khatoon and Kalam delineates curcumin dual blockade of NF-κB-driven neuroinflammation and Nrf2-mediated antioxidant defenses, yet its clinical utility has long been hamstrung by rapid hepatic metabolism. Khatoon and Kalam further highlight nanocarriers enhance curcumin brain bioavailability, positioning it as a viable alternative to conventional antiepileptics. Parallel work by Li et al. on (+)-borneol reveals a microglial reprogramming mechanism—TLR4-NFκB axis inhibition coupled with M1-to-M2 polarization shifts, mirroring Huang et al. findings with Gastrodia elata Blume extract in ischemia. Both studies highlight conserved anti-inflammatory pathways across distinct neuropathologies, suggesting broad applicability for terpenoid-rich botanicals. The frontier of neuroprotection embraces polyherbal strategies that mimic nature’s combinatorial logic. Next, Ren et al. pioneer a quadra-compound formulation (borneol, gastrodin, catalpol, and puerarin) for AD, achieving synergistic suppression of microglial hyperactivation and amyloid burden via TLR4/MyD88/NF-κB silencing. This phytochemical cocktail approach, leveraging additive pharmacokinetics and complementary targets, outperforms single-agent therapies and aligns with the multi-system etiology of neurodegeneration.

These advances underscore three pillars of progress—(1) Delivery innovation (intranasal niosomes, nanocarriers) to unlock the potential of poorly soluble phytochemicals; (2) Pathway convergence (NF-κB, Nrf2, TLR4) as a blueprint for target selection; and (3) Polypharmacology through rational multi-compound design. Future research may prioritize clinical translation of these platforms while exploring artificial intelligence-driven phytochemical screening and biomarker-guided personalized herbal regimens. By bridging ancient pharmacopeias with modern neurobiology, we may inch closer to therapies that are as complex and adaptive as the diseases they aim to treat.

### Nanotechnology and biomaterial-based approaches in neuroinflammation to engineer spinal cord injury

The fusion of nanotechnology and neurobiology is redefining regenerative medicine, offering precision-engineered solutions to combat neuroinflammation and neural degeneration. Amirian et al. pioneered this frontier with thymol-loaded poly (vinyl alcohol)/chitosan nanofibrous scaffolds for spinal cord injury (SCI) and strategically delivered Thymol *via* a biocompatible scaffold. The electrospun nanofibers provided structural support to bridge lesion gaps and also created a neurodegenerative niche. Their results underscore how biomaterial design can synergize with bioactive compounds to address SCI-related challenges such as physical disruption and chronic inflammation. Next, Bavandpouri et al. complement this approach by leveraging polydatin, to tackle its biochemical cascade.

Together, these studies exemplify two pillars of next-gen neurotherapy— (1) biomaterial scaffolds that remodel injury sites, and (2) small-molecule bioactives that quell inflammation. Future directions could integrate these strategies—e.g., embedding polydatin within nanofibrous matrices to achieve spatiotemporal control over drug release and tissue remodeling. Additionally, leveraging functionalized nanoparticles to target specific cell populations (e.g., reactive microglia) could amplify therapeutic precision. As the field evolves, combinatorial platforms that merge material science with immunometabolic modulation may hold the key to unlocking full functional recovery in SCI and beyond.

## Repurposing pharmaceuticals for neurodegenerative disorders: navigating promise and pitfalls

The repurposing of existing drugs for neurodegenerative diseases offers a pragmatic yet intricate path forward, demanding a balance between mechanistic insight and clinical nuance. A meta-analysis by Badawoud et al. reveals a critical divide—non-aspirin NSAIDs, with their COX-2 selectivity, may lower PD risk by tempering neuroinflammation, while aspirin and ibuprofen—agents with broader COX inhibition—fail to show benefit. This divergence underscores the importance of dissecting drug mechanisms beyond class-wide assumptions, emphasizing that even subtle differences in molecular targeting (e.g., COX-2 vs COX-1) can yield starkly divergent outcomes in neurodegeneration. A review of controversy around statins in Amyotrophic Lateral Sclerosis (ALS) is highlighted by Al-Kuraishy et al. to illustrate the precarious nature of repurposing. Statins’ dual role—neuroprotective in early stages *via* anti-inflammatory and lipid-lowering effects, yet potentially detrimental in advanced disease by impairing cholesterol-dependent repair—exposes the temporal fragility of therapeutic interventions. This paradox calls for a dynamic approach to treatment, where timing and genetic context (e.g., SOD1 mutations) dictate therapeutic candidacy, rather than a blanket application. Meanwhile, Dahalia et al. work with pirfenidone in epilepsy exemplifies the strategic repurposing by targeting the HMGB1/TLR4 axis (shared pathway in neuroinflammation and fibrosis), pirfenidone bridges disparate disease mechanisms, offering a blueprint for cross-disciplinary drug discovery. Such efforts highlight the value of prioritizing agents with pleiotropic anti-inflammatory or antioxidant properties that intersect with neurodegeneration’s multifactorial roots.

Here, the path forward lies in marrying mechanistic rigor with clinical adaptability. First, agents like pirfenidone, which act on convergent inflammatory nodes (e.g., DAMPs, TLR4), should be prioritized for their ability to disrupt multiple disease pathways. Second, therapies must be tailored to disease stages—statins may stabilize early ALS but harm late-stage patients, necessitating biomarker-guided timelines. Finally, patient stratification through genetic, metabolic, or inflammatory profiling could resolve conflicting trial outcomes, transforming repurposing from a gamble into a precision tool. Future studies should explore combinatorial regimens (e.g., COX-2 inhibitors paired with HMGB1 blockers) and leverage artificial intelligence to map off-target drug effects against neuroinflammatory networks, ensuring repurposed therapies are as nuanced as the diseases they aim to treat.

### Computational and mechanistic insights into targeted treatment

The integration of computational biology and mechanistic studies may revolutionize our ability to dissect neuroinflammatory cascades and pinpoint actionable therapeutic nodes. Saeed et al. explore network pharmacology and molecular dynamics simulations to unravel the fibro-inflammatory axis in Duchenne muscular dystrophy, identifying SMAD3 (transcriptional regulator of TGF-β signaling) as a central orchestrator of muscle-brain crosstalk. Their multimodal approach not only maps fibrosis-related pathways but also screens compounds for SMAD3 binding, offering a blueprint for repurposing pleiotropic phytochemicals against neuroinflammation-associated fibrosis. Further, in AD, Alrouji et al. employ a hybrid in silico-in vitro strategy to decode vanillin anti-inflammatory potential. Molecular docking and spectroscopic analyses reveal vanillin efficacy in stabilizing human transferrin by binding to its apically charged cleft. This structural stabilization can mitigate iron-mediated neurotoxicity and oxidative stress, while circular dichroism and *in vitro* assays confirm vanillin role in preserving transferrin functionality.

These studies underscore the transformative potential of merging computational rigor with mechanistic inquiry. Saeed et al. systems-level mapping of SMAD3-driven networks highlights the utility of multi-omics integration to identify upstream regulators of neuroinflammation. Meanwhile, Alrouji et al. focus on transferrin-vanillin interactions to illustrate how atomistic simulations can guide the rational design of small molecules to stabilize critical proteins in neurodegenerative milieus. Future efforts may explore molecular generative platforms to accelerate the discovery of SMAD3 inhibitors or transferrin-stabilizing agents while advancing digital twin models of neuroinflammatory cascades to predict therapeutic synergies. As computational tools evolve, their seamless integration with wet lab experimentation can be pivotal in translating mechanistic insights into therapies that disrupt neuroinflammation at its roots.

### Melatonin exerts neuroprotective effects by regulating circadian rhythms and neuroinflammation

Melatonin is emerging as a versatile modulator of neuroinflammatory cascades, offering therapeutic promise across neurodegenerative and environmental contexts. Gáll et al. illuminate its potential in AD, demonstrating that melatonin not only rescues cognitive deficits in rodent models but also dampens microglial hyperactivation. Although the precise mechanisms remain elusive, melatonin pleiotropic effects likely stem from its ability to scavenge reactive oxygen species, inhibit NLRP3 inflammasome assembly, and suppress pro-inflammatory cytokines. These actions position melatonin as a compelling adjunct to amyloid- or tau-targeted therapies by addressing both pathology and symptom burden. Further, Song et al. extend this narrative into the realm of environmental neurotoxicity, revealing how melatonin mitigates dim blue light-induced neuroinflammation which is a growing concern in our screen-saturated world. Their investigation uncovers a receptor-dependent mechanism, i.e., activation of MT2 receptors with melatonin suppresses NF-κB translocation, thereby blocking downstream inflammatory gene expression. This finding not only underscores melatonin role as a circadian synchronizer but also highlights its capacity to counteract externally triggered neuroinflammatory insults (e.g., light pollution). The study raises critical questions about modern environmental exposures and their disruption of endogenous neuroprotective rhythms.

Together, these studies reframe melatonin as a dual-axis therapeutic by balancing intrinsic circadian rhythms with extrinsic anti-inflammatory defenses. Findings from Gáll et al. suggest its utility in chronic neurodegeneration, while Song et al. emphasize its relevance in acute, environmentally driven inflammation. Future research can explore receptor-specific agonists (e.g., MT2-targeted compounds) to enhance precision, minimizing off-target effects linked to broad receptor affinity. Next, clinical trials may investigate melatonin synergy with lifestyle interventions (e.g., light exposure regulation) to amplify its circadian benefits. As the boundaries between environmental stressors and neurodegenerative processes blur, the multifaceted actions of melatonin offer a template for therapies that harmonize biological rhythms with neuroimmune resilience.

### Convergent innovation in neuroinflammation therapeutics

The rapidly expanding toolkit against neuroinflammation—spanning phytochemical pool bioenhancement, biomaterial engineering, drug repurposing, computational deconvolution, and circadian modulation—reflects a paradigm shift toward precision, synergy, and systems-level intervention. Natural compounds like quercetin and curcumin, once limited by bioavailability, now thrive through nanotechnology, while repurposed agents such as pirfenidone and statins are being recontextualized through biomarker-guided stratification. Computational platforms, exemplified by network mapping and compound(s)-protein(s) interaction modeling, are no longer auxiliary tools but drivers of mechanistic discovery, accelerating the transition from serendipity to rational design. Yet challenges persist—the duality of therapies like statins in ALS underscores the non-linear biology of neuroinflammation, demanding temporal precision and patient-specific adaptation. Similarly, the dual role of melatonin as a chronobiotic and NF-κB antagonist highlights the underappreciated interplay between environmental stressors and endogenous neuroprotection which is a frontier ripe for exploration. Now, the path forward lies in orchestrating these advances into context-aware therapeutic ecosystems. Imagine artificial intelligence-optimized polypharmacy regimens delivered via intranasal nanocarriers, timed to circadian peaks in neuroinflammation, while biomaterial scaffolds release polydatin at injury sites in response to real-time inflammatory biosensors. Such integration of computation, material science, and chronobiology could transform neuroprotection from reactive to proactive, targeting diseases at their inflammatory roots. Success will hinge on dismantling disciplinary silos, fostering partnerships between pharmacologists, computational biologists, and circadian neuroscientists to pioneer therapies as dynamic and resilient as the brain itself.

